# Mutanome and expression of immune response genes in microsatellite stable colon cancer

**DOI:** 10.18632/oncotarget.7293

**Published:** 2016-02-09

**Authors:** Rebeca Sanz-Pamplona, Raúl Gil-Hoyos, Adriana López-Doriga, M. Henar Alonso, Susanna Aussó, David G. Molleví, Cristina Santos, Xavier Sanjuán, Ramón Salazar, Ramón Alemany, Víctor Moreno

**Affiliations:** ^1^ Unit of Biomarkers and Susceptibility, Catalan Institute of Oncology (ICO), Bellvitge Biomedical Research Institute (IDIBELL) and CIBERESP, L'Hospitalet de Llobregat, Barcelona, Spain; ^2^ Translational Research Laboratory, Catalan Institute of Oncology (ICO), Bellvitge Biomedical Research Institute (IDIBELL), L'Hospitalet de Llobregat, Barcelona, Spain; ^3^ Department of Medical Oncology, Catalan Institute of Oncology (ICO), Bellvitge Biomedical Research Institute (IDIBELL), L'Hospitalet de Llobregat, Barcelona, Spain; ^4^ Pathology Service, University Hospital Bellvitge (HUB – IDIBELL), L'Hospitalet de Llobregat, Barcelona, Spain; ^5^ Department of Clinical Sciences, Faculty of Medicine, University of Barcelona (UB), Barcelona, Spain

**Keywords:** colorectal cancer, neoantigens, prognosis, antigen presentation, immune response

## Abstract

The aim of this study was to analyze the impact of the mutanome in the prognosis of microsatellite stable stage II CRC tumors. The exome of 42 stage II, microsatellite stable, colon tumors (21 of them relapse) and their paired mucosa were sequenced and analyzed. Although some pathways accumulated more mutations in patients exhibiting good or poor prognosis, no single somatic mutation was associated with prognosis. Exome sequencing data is also valuable to infer tumor neoantigens able to elicit a host immune response. Hence, putative neoantigens were identified by combining information about missense mutations in each tumor and HLAs genotypes of the patients. Under the hypothesis that neoantigens should be correctly presented in order to activate the immune response, expression levels of genes involved in the antigen presentation machinery were also assessed. In addition, CD8A level (as a marker of T-cell infiltration) was measured. We found that tumors with better prognosis showed a tendency to generate a higher number of immunogenic epitopes, and up-regulated genes involved in the antigen processing machinery. Moreover, tumors with higher T-cell infiltration also showed better prognosis. Stratifying by consensus molecular subtype, CMS4 tumors showed the highest association of expression levels of genes involved in the antigen presentation machinery with prognosis. Thus, we hypothesize that a subset of stage II microsatellite stable CRC tumors are able to generate an immune response in the host via MHC class I antigen presentation, directly related with a better prognosis.

## INTRODUCTION

Colorectal cancer (CRC) is a complex disease in which various types of molecular alterations are implicated [1]. Gene expression is largely deregulated [2], following diverse patterns that allow molecular subtyping into groups with diverse prognosis [3]. Stage I and II CRC patients have a moderate risk of relapse after surgical resection, whereas stage III patients have a higher chance of recurrence. Taking advantage of genome-wide techniques, a large number of studies have performed gene expression profiling to identify molecular prognosis biomarkers useful to discriminate between good and poor prognosis CRC tumors [4, 5], although no marker has been yet adopted in routine clinical practice [6]. In addition to gene expression, diverse aberrations at DNA level have been widely described to contribute to CRC progression such as copy number aberrations, point mutations or altered methylation [7, 8]. Also, increasing data support the main role of tumor microenvironment in CRC progression and prognosis [9, 10]. Tumor microenvironment is composed by a heterogeneous population of stromal cells such as fibroblasts, extracellular matrix components, and also immune cells [11]. Indeed, evasion of immune surveillance and/or suppression of immune system have been described as a hallmark of cancer [12].

The cellular machinery is able to recognize mutant proteins and cleave them via the proteasome. Then, the generated peptides bind to MHC molecules to be presented to T cells [13]. In cancer cells, somatic mutations generate cancer-specific antigens that could be targeted [14]. In fact, an association between HLA expression and prognosis has been described in various types of cancer, including CRC [15]. Immune system activation has been specifically recognized in CRC tumors with DNA mismatch repair deficiencies such as microsatellite instable (MSI) CRC, that accumulate an elevated number of point mutations [16]. The high mutational load in MSI tumors creates many tumor-specific neoantigens, typically 10 to 50 times more than microsatellite stable (MSS) tumors [17]. Indeed, it has been proposed that the higher level of neoantigens and lymphocytic reaction in MSI tumors may contribute to a better patient survival [18]. The MSI subset of colorectal tumors is therefore a good candidate for checkpoint immunotherapy [19], however not as much research has been devoted to study the role of immune system activation in response to mutations in the subset of MSS tumors. Even for MSS tumors, a high number of neoepitopes could be associated a better prognosis.

Exome sequencing has been revealed as a useful technique for mutation discovery in cancer [20, 21]. This technique is also useful to predict, from DNA mutations, putative short peptides (neopeptides) capable to elicit immunoreactivity through their presentation at the cell surface as major histocompatibility complex (MHC) ligands. Recently, our group performed an exome sequencing analysis of a homogenous group of microsatellite stable (MSS) stage II colorectal tumors and described the landscape of somatic mutations [22]. Here we aim to compare the mutational profile of good and poor prognosis stage II MSS CRC tumors, and to explore whether the activation of the immune system in response to somatic mutations is related to prognosis.

## RESULTS

### No significant association was found between single nucleotide variants (SNVs) and prognosis

**Figure 1:**
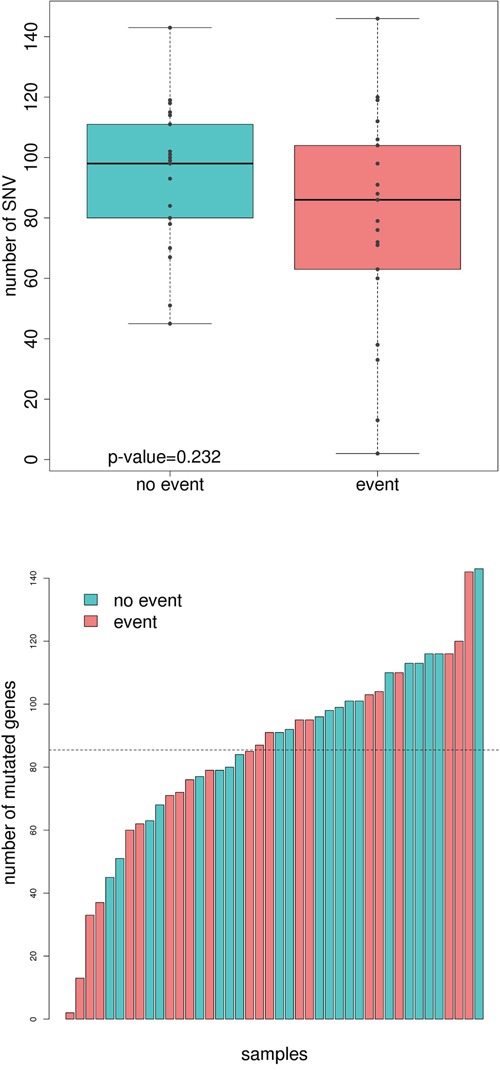
Comparison of total number of mutations and mutated genes between good and poor-prognosis tumors **A.** Boxplot showing the number of mutations in the group of tumors with no event (turquoise) and event (light red). **B.** Number of mutated genes in each sequenced sample. Those tumors from patients with no event are painted in turquoise whereas the ones who relapse are painted in light red.

A total of 3,597 potentially functional single nucleotide variants (SNVs) were found across the 42 sequenced tumors. No differences on the total number of mutations between good and poor prognosis groups were detected (Figure 1A). When SNVs were classified by functional effect, only “missense near splice site” mutations were marginally significant (*P*=0.04, Supplementary Figure 1). Good prognosis tumors accumulated 1961 SNVs (median 98, range 45 to 153) whereas relapsing tumors accumulated 1675 SNVs (median 86, range 2 to 146). The median difference was 12 (Mann-Whitney Test p-value=0.23). From these, only 23 were recurrent (found in at least 2 tumors): 16 were shared by good and poor prognosis groups, 4 were good-exclusive (2 located in *WASL* gene, 1 in *ERP27* and 1 in *C3orf27*) and 3 were poor-exclusive (located in *AGTRAP*, *SYNPO2*, and *APC* genes). No single mutation was associated with prognosis, though the low frequency of recurrent mutations derived in a low power to detect specific differences.

### Prognosis association of SNVs cumulated by genes, pathways, and their position into the human interactome

SNVs were mapped into genes harboring them. The average number of mutated genes per sample was 83 (range: 2-136). In line with SNV results, no differences between the total number of mutated genes between good and poor prognosis groups were detected (Figure 1B). The good prognosis tumors had 1618 mutated genes (median 96, range 45 to 143) whereas the tumors with worse prognosis accumulated 1426 (median 85, range 2 to 142, median difference 11, Mann-Whitney test p-value=0.21). Both groups shared 270 genes (8%), when the expected was less than 1%, so they were probably related to common pathways in carcinogenesis. Regarding recurrently and exclusively mutated genes, 12 genes were found to be mutated in more than 3 poor-prognosis tumors including *BRAF* and *POLE*. On the other hand, good-prognosis tumors had 15 exclusively-mutated genes including *NOTCH3* (Table 1). Not surprisingly, the well-known *APC*, *TP53*, *KRAS*, along with *FAT4*, *FBXW7*, and *RYR3* were equally mutated in both groups.

**Table 1:** Recurrently and exclusively mutated genes

Good prognosis			
Gene	# Tumors	Description	Reported in literature
*DNHD1*	4	Involved in microtubule motor activity	---
*PCDH17*	4	Potential calcium-dependent cell-adhesion protein	Hu X et al. Protocadherin 17 acts as a tumour suppressor inducing tumour cell apoptosis and autophagy, and is frequently methylated in gastric and colorectal cancers. J Pathol. 2013 Jan;229(1):62-73.
*USH2A*	4	Involved in hearing and vision	Kim N et al. Somatic mutaome profile in human cancer tissues. Genomics Inform. 2013 Dec;11(4):239-44. doi: 10.5808/GI.2013.11.4.239. Epub 2013 Dec 31.
*ADCY2*	3	Membrane-bound, calmodulin-insensitive adenylyl cyclase	Fang LT et al. Comprehensive genomic analyses of a metastatic colon cancer to the lung by whole exome sequencing and gene expression analysis. Int J Oncol. 2014 Jan;44(1):211-21.
*ATP1B1*	3	ATPase activator activity	Selvakumar P et al. Epigenetic silencing of Na, K-ATPase β 1 subunit gene ATP1B1 by methylation in clear cell renal cell carcinoma. Epigenetics. 2014 Apr;9(4):579-86.
*CAD*	3	This protein is a “fusion” protein encoding four enzymatic activities of the pyrimidine pathway	---
*ELFN1*	3	Postsynaptic protein that regulates circuit dynamics in the central nervous system	---
*ERP27*	3	Endoplasmic reticulum resident protein 27	---
*ESR1*	3	Nuclear hormone receptor	Caiazza F, et al. Estrogen receptors and their implications in colorectal carcinogenesis. Front Oncol. 2015 Feb 2;5:19.
*F7*	3	Coagulation factor	Naderi A. Coagulation factor VII is regulated by androgen receptor in breast cancer. Exp Cell Res. 2015 Feb 1;331(1):239-50.
*ITIH1*	3	May act as a carrier of hyaluronan in serum or as a binding protein between hyaluronan and other matrix protein	Hamm A et al. Frequent expression loss of Inter-alpha-trypsin inhibitor heavy chain (ITIH) genes in multiple human solid tumors: a systematic expression analysis. BMC Cancer. 2008 Jan 28;8:25.
*LHX5*	3	Plays an essential role in the regulation of neuronal differentiation and migration	---
*NFATC1*	3	Plays a role in the inducible expression of cytokine genes in T-cells	Tripathi MK et al. Nuclear factor of activated T-cell activity is associated with metastatic capacity in colon cancer. Cancer Res. 2014 Dec 1;74(23):6947-57.
*NOTCH3*	3	Functions as a receptor for membrane-bound ligands Jagged1, Jagged2 and Delta1 to regulate cell-fate determination	Wang XW et al. MicroRNA-206 attenuates tumor proliferation and migration involving the downregulation of NOTCH3 in colorectal cancer. Oncol Rep. 2015 Mar;33(3):1402-10.
*PTPRS*	3	Receptor-type tyrosine-protein phosphatase implicated in cell adhesion	Wang ZC et al. PTPRS Acts as A Metastatic Suppressor in Hepatocellular Carcinoma by Control of EGFR Induced Epithelial-Mesenchymal Transition. Hepatology. 2015 May 22.
**Poor prognosis**			
**Gene**	**# Tumors**	**Description**	**Reported in literature**
*CSMD1*	5	Membrane receptor	Farrell C et al. Somatic mutations to CSMD1 in colorectal adenocarcinomas. Cancer Biol Ther. 2008 Apr;7(4):609-13.
*BRAF*	3	Protein kinase involved in the transduction of mitogenic signals from the cell membrane to the nucleus.	Clancy C et al. *BRAF* mutation is associated with distinct clinicopathological characteristics in colorectal cancer: a systematic review and meta-analysis. Colorectal Dis. 2013 Dec;15(12):e711-8.
*CACNA1G*	3	Involved in a variety of calcium-dependent processes, including gene expression, cell motility, cell division and cell death	Berg M et al. Molecular subtypes in stage II-III colon cancer defined by genomic instability:early recurrence-risk associated with a high copy-number variation and loss of RUNX3 and CDKN2A. PLoS One. 2015 Apr 16;10(4):e0122391.
*CCDC168*	3	Coiled-coil domain-containing protein 168	---
*DPYD*	3	degradation of the chemotherapeutic drug 5-fluorouracil	Innocenti F. DPYD variants to predict 5-FU toxicity: the ultimate proof. J Natl Cancer Inst. 2014 Nov 7;106(12).
*DSCAML1*	3	Cell adhesion molecule that plays a role in neuronal self-avoidance.	---
*NRG1*	3	Transcriptional repressor that binds NRG1 response elements (NRE) of target promoters	Lim B, et al. Genome-wide mutation profiles of colorectal tumors and associated liver metastases at the exome and transcriptome levels. Oncotarget. 2015 Jun 1.
*PAPPA2*	3	Metalloproteinase which specifically cleaves IGFBP-5	---
*POLE*	3	Participates in DNA repair and in chromosomal DNA replication	Haraldsdottir S, et al. Colon and endometrial cancers with mismatch repair deficiency can arise from somatic, rather than germline, mutations. Gastroenterology. 2014 Dec;147(6):1308-1316.
*ROBO2*	3	Receptor implicated in cellular migration	Je EM, et al. Frameshift mutations of axon guidance genes ROBO1 and ROBO2 in gastric and colorectal cancers with microsatellite instability. Pathology. 2013 Dec;45(7):645-50.
*SLITRK5*	3	Suppresses neurite outgrowth	---
*SPTA1*	3	The major constituent of the cytoskeletal network underlying the erythrocyte plasma membrane	Iwakawa R, et al. Expression and clinical significance of genes frequently mutated in small cell lung cancers defined by whole exome/RNA sequencing. Carcinogenesis. 2015 Jun;36(6):616-21.

Next, a pathway enrichment analysis was performed, stratified by prognosis group. Both groups were enriched in mutated genes that mapped to pathways classically related to cancer, like p53 signaling or Wnt pathway. However, tumors in poor prognosis group had more mutations in pathways related to DNA repair and polymerase activity whereas good prognosis tumors had more mutations in genes related to cell cycle or apoptosis, among others (Table 2 and Supplementary Table 1).

**Table 2:** Mutated pathways and functions in each group of patients

Good prognosis				
FUNCTION	score good	score poor	dif.score	p-val
APOPTOSIS (KEGG)	0.903	0.432	−0.472	0.001
SMALL CELL LUNG CANCER (KEGG)	0.828	0.452	−0.376	0.012
DILATED CARDIOMYOPATHY (KEGG)	0.837	0.540	−0.297	0.039
VEGF SIGNALING PATHWAY (KEGG)	0.817	0.538	−0.279	0.037
CELL CYCLE (KEGG)	0.621	0.365	−0.256	0.020
LIPID KINASE ACTIVITY (GO)	1.863	0.487	−1.376	0.002
INOSITOL OR PHOSPHATIDYLINOSITOL KINASE ACTIVITY (GO)	1.242	0.325	−0.917	0.002
**Poor prognosis**				
**FUNCTION**	**score good**	**score poor**	**dif.score**	**p-val**
NUCLEOTIDE EXCISION REPAIR (KEGG)	0.113	0.398	0.285	0.050
TIGHT JUNCTION (KEGG)	0.389	0.654	0.265	0.019
DNA POLYMERASE ACTIVITY (GO)	0.000	0.812	0.812	0.008
DNA DIRECTED DNA POLYMERASE ACTIVITY (GO)	0.000	0.626	0.626	0.008

Finally, genes harboring mutations were mapped into the human interactome and analyzed to test the hypothesis that relevant genes might have central positions in networks (i.e act as hubs). However, no significant differences were found in the centrality measures degree (*P*=0.65), betwenness (*P*=0.47), closeness (*P*=0.56), and eccentricity (*P*= 0.38), between the two groups of tumors.

### Number of neoantigens and expression levels of genes involved in antigen presentation machinery is associated with prognosis, and this association changes within molecular subtypes

**Figure 2:**
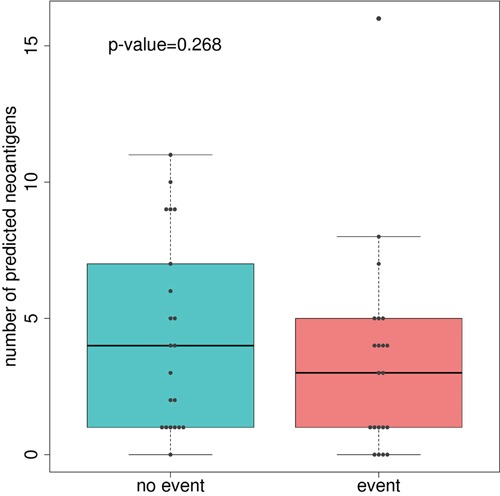
Boxplot comparing the number of predicted neoantigenes between good and poor-prognosis tumors P-value was calculated using Man-Whitney U test.

**Figure 3:**
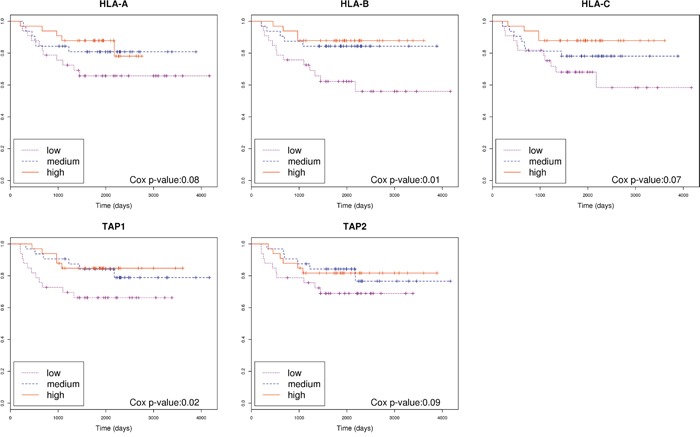
Association of genes related to antigen presentation machinery with prognosis Kaplan-Meier curves separating patients based on the level of expression of genes *HLA-A*, *HLA-B*, *HLA-C*, *TAP1* and *TAP2*. Cox p-value was calculated for each gene.

Somatic missense mutations can activate T cytotoxic responses. Thus, we used bioinformatics tools to predict putative neoantigens by combining information about HLA genotypes and missense mutations in each patient. Then, we assessed if a relationship existed between the number of hypothetical neoantigens generated by the tumor and the patients' outcome. No significant differences between good and poor prognosis groups of tumors were found (Figure 2), even if the level of expression of genes harboring missense mutations were taken into account (Supplementary Figure 2). However, a trend was identified when extremes were compared: 4 out of 5 tumors with 0 predicted neoantigens had relapsed whereas 5 out of 6 tumors with more than 8 predicted neoantigens had not relapsed. We were intrigued by the relapse of the tumor with the highest number of predicted neoantigens (n=16). Taking advantage of gene expression data, we realized that this tumor had a marked under-expression of indispensable genes for the neoantigen presentation such as *HLA-A*, *HLA-B*, *HLA-C*, *TAP1*, and *TAP2*. Consequently, under the hypothesis that neoantigens only activate immune response if correctly presented by antigen presentation machinery, the levels of expression of these genes were analyzed in the complete sample of 98 tumors of the Colonomics study with expression data that included those 42 analyzed by NGS. Indeed, gene expression levels of genes related to antigen presentation were associated with prognosis. Poor outcome was observed for tumors under-expressing *HLA-B* (*P*=0.01), and *TAP1* (*P*=0.02) as shown in Figure 3. Genes of HLA class II (*HLA-DPA1*, *HLA-DPB1*, *HLA-DQA1*, and *HLA-DQB1*) were also analyzed, although no association with prognosis was found (Supplementary Figure 3). For a more comprehensive analysis of the role of immune response genes, we used all these variables to perform a principal component analysis (PCA). As a result, two extreme group of samples emerged. On one hand, patients with poor prognosis under-expressed HLA and TAP genes (regardless of the number of neoantigens). On the other hand, samples with good prognosis over-expressed HLAs and contained higher levels of predicted neoantigens (Supplementary Figure 4).

**Figure 4:**
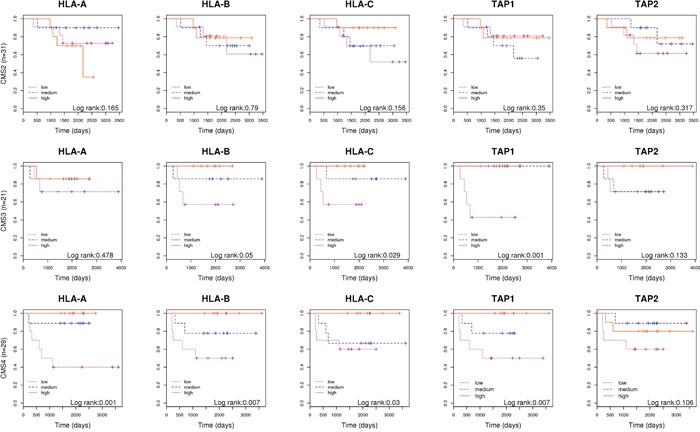
Molecular subtyping Kaplan-Meier curves separating patients based on the level of expression of genes *HLA-A, HLA-B, HLA-C, TAP1* and *TAP2*; in tumors classified as CMS2, CMS3 and CMS4 separately.

Recently, a group of experts has reported a robust classification of CRC for future clinical stratification that group tumors in four subtypes based on common molecular and clinical characteristics [3]. Under the hypothesis that our results could vary within subtypes, we assessed the prognosis value of genes related with antigen presentation stratifying by consensus molecular subtypes. We found that the expression of genes of immune response were not associated to prognosis for tumors classified as CMS2 “canonical” subtype. However, the association was very strong among the CMS4 “mesenchymal” and in CMS3 “metabolic” and subtypes. Tumors overexpressing genes involved in antigen presentation in these subtypes had better prognosis (Figure 4). Of note, because all our tumors were MSS, only 6 tumors were classified as CMS1 “MSI subtype” and were not analyzed, although 2 of them had relapsed.

### Good-prognosis tumors showed higher levels of lymphocytic infiltration

**Figure 5:**
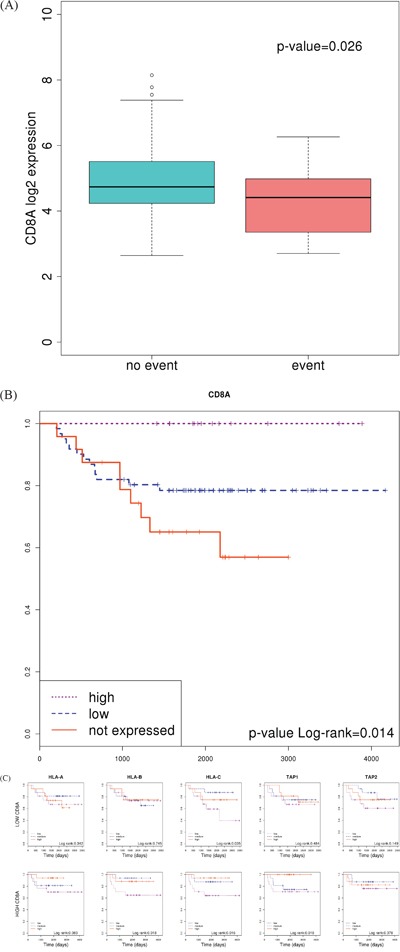
Association of *CD8A* expression with prognosis **A.** Boxplot showing differences between patients who experienced relapse or not. P value was calculated using a t-test **B.** Kaplan-Meier curve separating a cluster of patients of good prognosis who over-express *CD8A*. **C.** Kaplan-Meier curves separating patients based on the level of expression of genes *HLA-A, HLA-B* and *TAP1*; in tumors classified as “high CD8A” and “low CD8A”, separately.

Apart from neoantigen generation and presentation, a third player is necessary to activate an immune response against the tumor cell: T-cell receptors should bind to MHC-antigen complexes. Under this hypothesis, the level of expression of *CD8A* was used as a marker of T-cell infiltration and compared between the two groups of tumors [28]. CD8 is a specific marker for T-cells binding MHC Class I but not MHC Class II. As expected, good prognosis tumors showed higher levels of this marker than poor prognosis ones (p-value=0.026) suggesting that not only antigen presentation but also proper recognition by T-cells is necessary for the host immune system to attack the tumor (Figure 5A). Indeed, a Kaplan-Meier curve plotting patients classified on the basis of *CD8A* expression (Supplementary Figure 5) clearly identified a group of 13 patients over-expressing the gene who did not experience relapse (Figure 5B).

Based on this result, we stratified patients into “high” and “low” groups according to *CD8A* expression. Then, we looked for the prognosis value of HLAs and TAP genes in each group. As expected, survival curves were significantly different in those patients showing T-cell infiltration (high *CD8A* levels); whereas no differences were found in those patients without or with low T-cell infiltration (Figure 5C). This result suggested that, in order to be recognized and eliminated by the immune system, T-cells needs to infiltrate the tumor (indicated by high *CD8A* levels).

**Figure 6:**
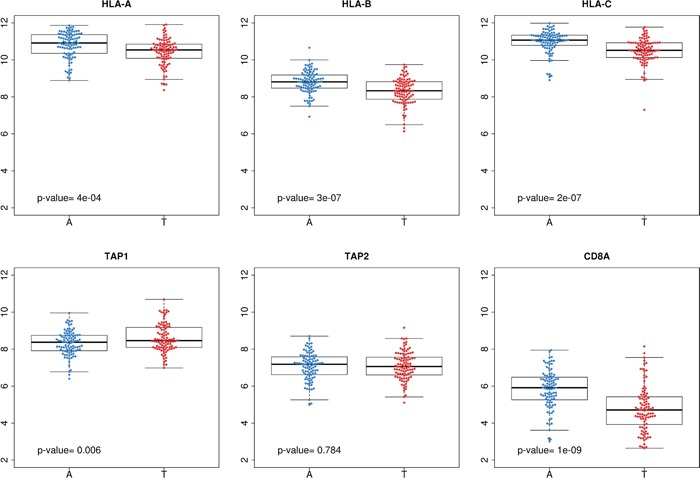
Comparison between tumor and adjacent normal mucosa Gene expression levels of HLA class I genes, *TAP1*, *TAP2* and *CD8A* in adjacent mucosa (blue) and paired tumor tissue (red).

Based on this, we hypothesized that colon tumors evade immune responses underexpressing genes involved in antigen presentation and/or preventing the infiltration of T-cells into the tumor bulk. Indeed, an analysis comparing the levels of expression of MHC class I and TAPs genes, as well as *CD8A* gene, showed that, compared with adjacent normal mucosa, tumors underexpressed HLA genes (*P*=4e-4 for HLA-A, *P*=3e-7 for HLA-B, and *P*=4e-5 for HLA-C). *CD8A* was also underexpressed (*P*=1e-9), but not TAP1 nor TAP2 genes (Figure 6).

## DISCUSSION

Our study has shown that there are no major differences between the exome mutational landscape of good and poor-prognosis group of tumors. However, our analyses revealed that tumors able to properly present neoantigens and showing T-cell infiltration have better prognosis. Indeed, increasing data support the idea that the endogenous T cell compartment is able to recognize peptide epitopes that are displayed by major histocompatibility complexes (MHCs) on the surface of the tumor cells [29]. We searched for patient-specific neoantigens that might be generated from both driver and passenger mutations acquired during tumorigenesis. Then, we tested the hypothesis that recognition of such neoantigens by the host immune cells could influence patients' survival. Certainly, our results pointed to a better outcome in those patients not only with a higher number of neoantigens (although not statistically significant, a trend was observed), but also over-expressing genes involved in antigen presentation machinery. In agreement with our results, an association between the number of predicted neoantigens in a tumor with increased patient survival has been recently described in a meta-analysis including six different types of tumors [30]. Thus, we hypothesize that poor-prognosis MSS stage II CRC tumors are unable to generate an immune response in the host via MHC class I antigen presentation.

It is well known that HLA class I functional abrogation represents a mechanism by which tumors circumvent immune surveillance [31]. In CRC, HLA class I loss has been described as a more frequent event in MSI tumors than in MSS tumors [32]. Regarding non-classical HLA class I molecules, their over-expression is one of the immunosuppressive strategies used by tumors [33]. Zeestraten et al. postulated that patients whose CRC tumors showed loss of HLA-E and HLA-G had better overall survival [15]. However, we have not reproduced this result in our data. We have found that expression of *CD8A*, considered a marker of lymphocyte infiltration, is higher in patients with better prognosis, probably indicating an activation of immune response against the tumor. This result was concordant with other studies correlating CD8+ lymphocytic infiltration with better survival in colon cancer [34, 35]. In addition, a stratification of our samples into “high CD8A” and “low CD8A” showed that levels of HLA and TAP1 genes are only relevant predicting survival in “high CD8A” tumors. This leads us to the conclusion that proper antigen presentation is only relevant for the inhibition of growth in those tumors showing T-cell infiltration. Indeed, it has been recently reported that loss of tapasin correlates with a reduction in CD8+ t-cell immunity and is associated with tumor progression in CRC [36]. It is important to note that patients in our series had not been treated with chemotherapy. In consequence, we consider that our results mirror the original response of the patients against the residual disease without treatment perturbing the host immune response.

Tumor-specific antigens that are generated by somatic mutation –neoantigens- can influence patient response to immunotherapy treatment and contribute to tumor reduction [37]. It is well known that microsatellite instable (MSI) CRC tumors are frequently characterized by inflammatory lymphocytic infiltration which is associated with a better outcome than microsatellite stable (MSS) CRC, probably reflecting a more effective immune response [38]. Although lymphocyte infiltration is characteristic of MSI tumors, it also has been shown to predict better prognosis in MSS tumors [39-41]. It is important to highlight that in this work all the analyzed CRC tumors are MSS, and a weak immune response should be expected, al least in comparison with MSI tumors. Nevertheless, a recent work assessing immunogenicity of somatic mutations in gastrointestinal cancers demonstrated that a clinically relevant anti-mutation T cell response could be also triggered against tumors with a low mutational load [42]. Thus, we postulate that MSS tumors with high HLA, mutational load and antigen processing pathways acquire a phenotype that helps cancer cells to evade the immune system of the host. Indeed, a comparison between tumor and adjacent normal mucosa revealed that tumors significantly underexpressed HLA class I genes and CD8A, suggesting a poorer ability for antigen presentation and recognition.

We were interested in analyzing our data according to the recent proposal of Consensus Molecular Subtypes that classifies CRC tumors into CMS1 “MSI immune”, CMS2 “Canonical”, CMS3 “Metabolic” and CMS4 “Mesenchymal” [3]. While tumors belonging to CMS1 (not included in our dataset) are more prone to induce a host immune response, our results showed that CMS3 and especially CMS4 tumors might also elicit an immune response. In agreement, recent studies conclude that CMS4 “mesenchymal” phenotype is characterized by a high stromal infiltration [43, 44]. This subgroup usually has worse prognosis than others. So, this stratification inside subgroups might be useful to further selection of patients requiring more aggressive or specific therapies in CRC. It is also important to note that the CMS2 “canonical” subtype comprising almost 40% of CRC tumors seems not to be able to elicit an immune response.

Although no single SNV was found to be associated with prognosis, several genes have been found to be exclusively and recurrently mutated in the poor-prognosis group of tumors such as classically CRC related genes *POLE* and *BRAF*. Mutations in *POLE* have been related to shorter patient survival [45]. Also in agreement with our results, *BRAF* mutations have been associated with a decrease in survival rate in CRC patients [46-48] and specifically in MSS CRC tumors [49]. We have found *CACNA1G* missense mutations in three tumors, all of them exhibiting poor-prognosis. Interestingly, a recent paper by Berg et al. reported that losses of *CACNA1G* by copy number changes were associated with early recurrence [50]. Thus, we hypothesized that loss of this gene could be a poor prognosis biomarker. Regarding functions in which mutated genes are implicated, several pathways have been found exclusively mutated in good and poor group of tumors. This result suggests that diverse molecular alterations could converge in similar phenotypes necessary for cancer progression and invasion.

This study also has several limitations. Besides the limited sample size, we have not a direct evidence that the mutated proteins generate neoantigens, but have used instead bioinformatics prediction tools. Moreover, it has been reported that HLA inference program HLAMiner achieves a good performance with RNA-seq or whole genome data rather than with exome sequencing data [26]. Finally, it is well known that each gene could codify several protein isoforms. However, in this work, to predict neoantigens only those isoforms reported in UniProt repository as the most common ones have been taken into account. Also, tumor infiltrating lymphocytes have not been measured directly, but CD8A expression has been used as a surrogate variable.

In conclusion, this analysis of prognosis in relation to somatic mutations in CRC has shown that although no single mutation was significantly associated with prognosis, some genes were found to be exclusively mutated in poor-prognosis tumors. Moreover, neither the total number of mutations nor the number of predicted neoantigens were different between good and poor-prognosis groups of tumors. However, those patients with higher levels of *CD8A* (as a marker of lymphocytic infiltration) and HLA-class I genes expression showed better prognosis. These results pave the way for future studies assessing the putative role of genes implicated in tumor-specific antigen presentation and recognition as prognostic biomarkers in pre-metastatic tumors, specifically in the subset of tumors classified as “metabolic” or “mesenchymal”. In addition, they suggest that personalized immunotherapy could stand for a treatment opportunity in patients suffering MSS CRC tumors.

## MATERIALS AND METHODS

### Patients and samples

This study included a subset of 42 paired adjacent normal and tumor tissues (84 samples) from a previously described set of 100 patients with colon cancer diagnosed at stage II and microsatellite stable tumors (colonomics project –CLX-: www.colonomics.org; NCBI BioProject PRJNA188510). The series of 42 patients was selected to include the 21 patients who experienced relapse and 21 without metastatic progression after a minimal follow-up of 3 years (Supplementary Table 2). None of them received chemotherapy. All patients were recruited at the Bellvitge University Hospital (Barcelona, Spain). Written informed consent was obtained from all patients and the Institution's Ethics Committee approved the protocol. DNA was extracted using a standard phenol-chloroform protocol. Moreover, gene expression profiles of these tumors have been performed [23] and is available in GEO repository (GSE44076).

### Exome sequencing and somatic single nucleotide (SNV) variants selection

Exome sequencing pipeline was extensively described in a previous work [22]. Briefly, Genomic DNA from the set of 42 adjacent-tumor paired samples was sequenced in the National Center of Genomic Analysis, Barcelona, Spain (CNAG) using the Illumina HiSeq-2000 platform. Exome capture was performed with the commercial kit Sure Select XT Human All Exon 50MB (Agilent). After data alignment and processing, high quality single nucleotide variants (SNVs) were identified using GATK software and germline variants were filtered. Finally, somatic SNVs were annotated using the SeattleSeq Variant Annotation web tool. Only potentially functional single nucleotide variants (SNVs) were taken into account in this work. Those include variants annotated as: *coding-synonymous-near-splice*, *missense*, *missense-near-splice*, *splice-3*, *splice-5*, *stop-gained*, *stop-gained-near-splice*, *stop-lost*, *utr-3* and *utr-5*.

### Functional analysis

Gene sets containing function and pathway information from “KEGG”, “Biocarta”, “Reactome”, and “GO” were downloaded from the Molecular Signatures Database [24]. For each gene set, an enrichment score was calculated for each tumor by dividing the number of mutations mapping into genes constituting the gene set by the number of genes in such gene set. The score was then compared for good and poor group of tumors. To assess if differences in scores were significant, a p-value was calculated by randomly permuting the tumor group label. The number of permutations was calculated in each case to ensure that the minimum p-value was at least as small as required by the Bonferroni correction at nominal 0.05 significance level.

### Interactome analysis

Human pairs of protein-protein interactions were downloaded from Hippie database [25] (last updated: 09/05/14). Only highly reliable interactions were taken into account (score > 0.72) to construct a human protein-protein interaction network containing 9,963 nodes and 49,847 edges. This network was used as a framework in which mutated genes were mapped. To each mutated gene in each tumor, centrality parameters degree, betweenness, closeness and eccentricity were calculated using igraph library in R.

### HLA genotyping and neoantigens prediction

Exome sequencing data from normal samples was used to infer MHC class I alleles of HLA-A and HLA-B (genes that predominantly mediates anti-tumor response) using HLAminer software [26].

FASTA sequences extracted from UniProt were used to translate information about DNA missense mutations into an aminoacid change level (only isoforms 1 were taken into account). For each missense mutation, a sequence of 21-aminoacids centered on the mutation and the corresponding wild type peptide were analyzed for potential neoantigens. NetMHCpan 2.8 Server [27] was used to infer putative immunogenic peptides (8 to 11 aminoacids) combining information about HLAs genotypes and peptides harboring missense mutations. An immunogenic epitope was defined as a mutated peptide with high affinity for one HLA allele of the patient (IC50<50nM) and low affinity for the wild-type counterpart (IC50>500nM). Information about the level of expression of each gene generating an immunopeptide was also computed.

### Survival analyses

Kaplan–Meier curves were plotted to represent the results, dividing the range of gene expression into quartiles and log-rank test were computed. Also, Cox models were fitted to consider multiple variables simultaneously and control for potential confounding.

### Molecular subtyping

The CMSclassifier R package [3] was used to classify our samples into the four CRC consensus molecular subtypes CMS1, CMS2, CMS3 and CMS4, using a Random Forest approach. Also, a principal components analysis (PCA) was performed to explore dispersion in our data based on the number of predicted neoantigens and expression level of genes of interest.

## SUPPLEMENTARY FIGURES AND TABLES




