# Supplementary material for: Mutanome and expression of immune response genes in microsatellite stable colon cancer
Source: Oncotarget. 2016 Feb 9;7(14):17711–25. doi: 10.18632/oncotarget.7293 (PMC4951244; doi:10.18632/oncotarget.7293)
Supplement: Supplementary file 1 [file oncotarget-07-17711-s001.pdf]

SUPPLEMENTARY FIGURES AND TABLES

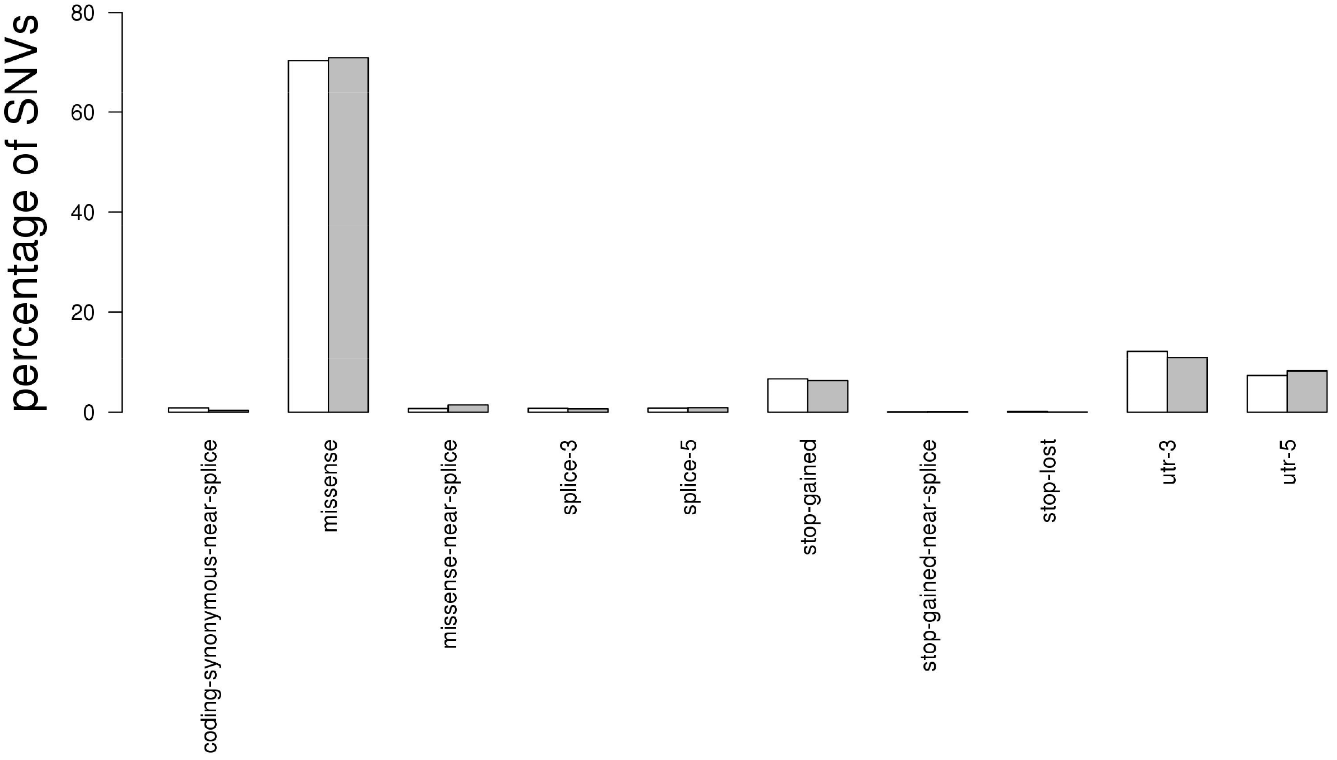

Supplementary Figure 1: Barplot showing the number of mutations in the group of tumors with no event (white) and event (grey), stratifying by type of mutation.

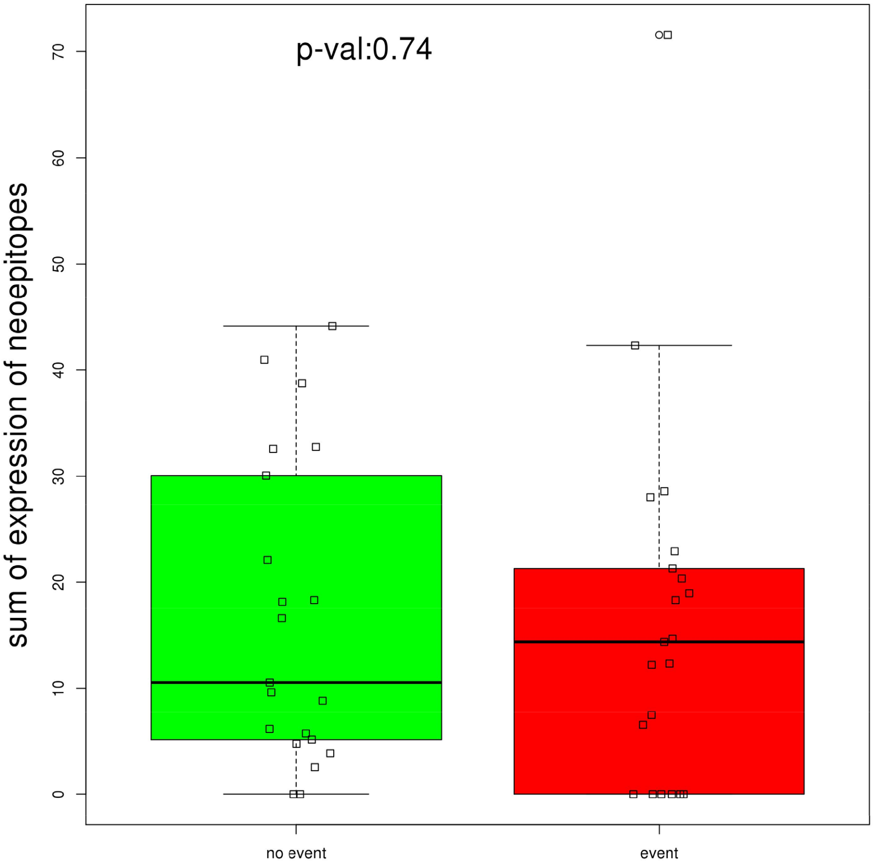

Supplementary Figure 2: Boxplots showing differences in mean of expression levels of genes harbouting mutations predicted go generate neoantigens, between good and poor-prognosis group of tumors.

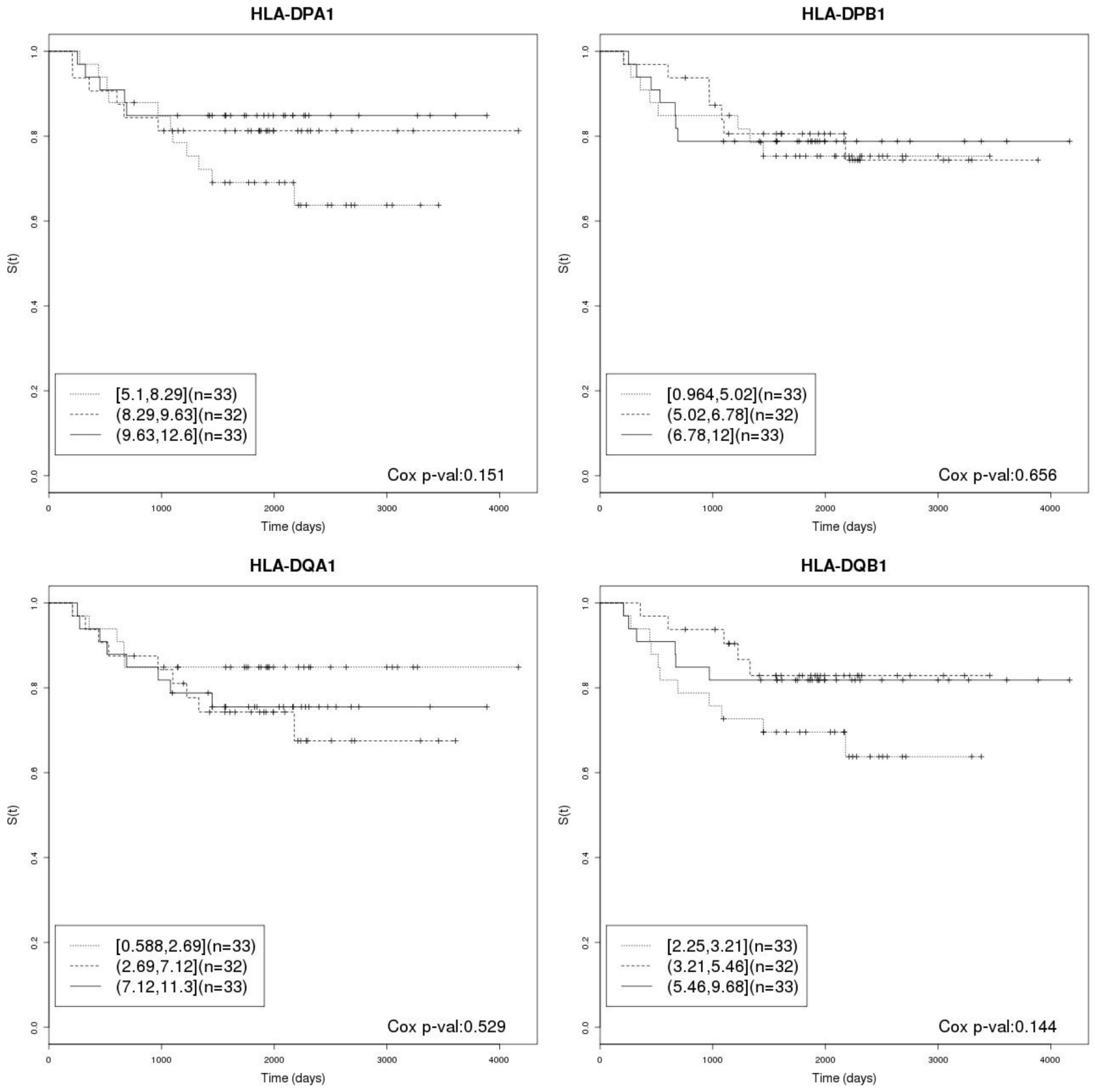

Supplementary Figure 3: Kaplan-Meier curves showing MHC class II genes association with prognosis.

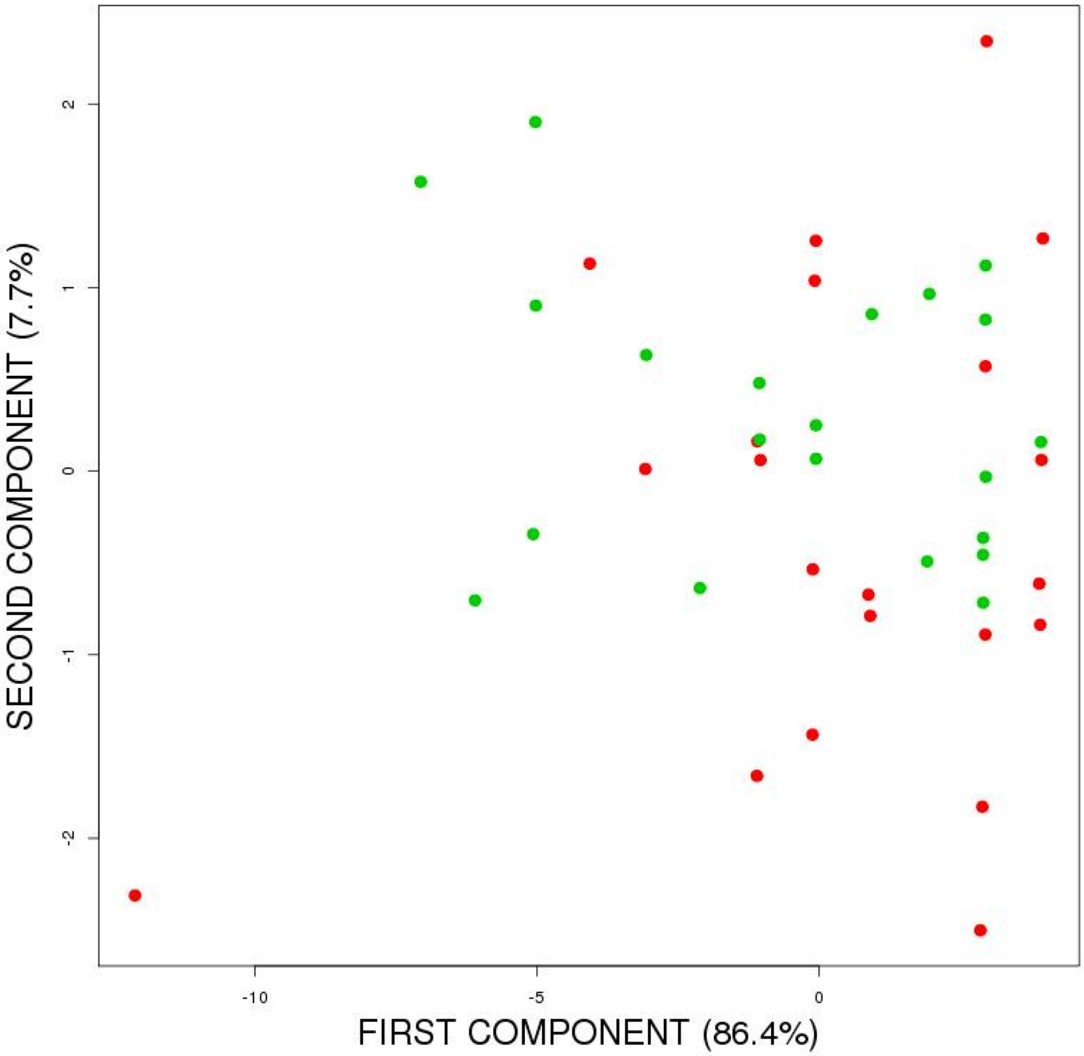

Supplementary Figure 4: PCA scatter plot representing the dispersion of the samples based on number of predicted neoepitopes and gene expression levels of HLAs and TAPs. Poor-prognosis tumors (red) and good-prognosis tumors (green) were plotted in 1st and 2nd principal components.

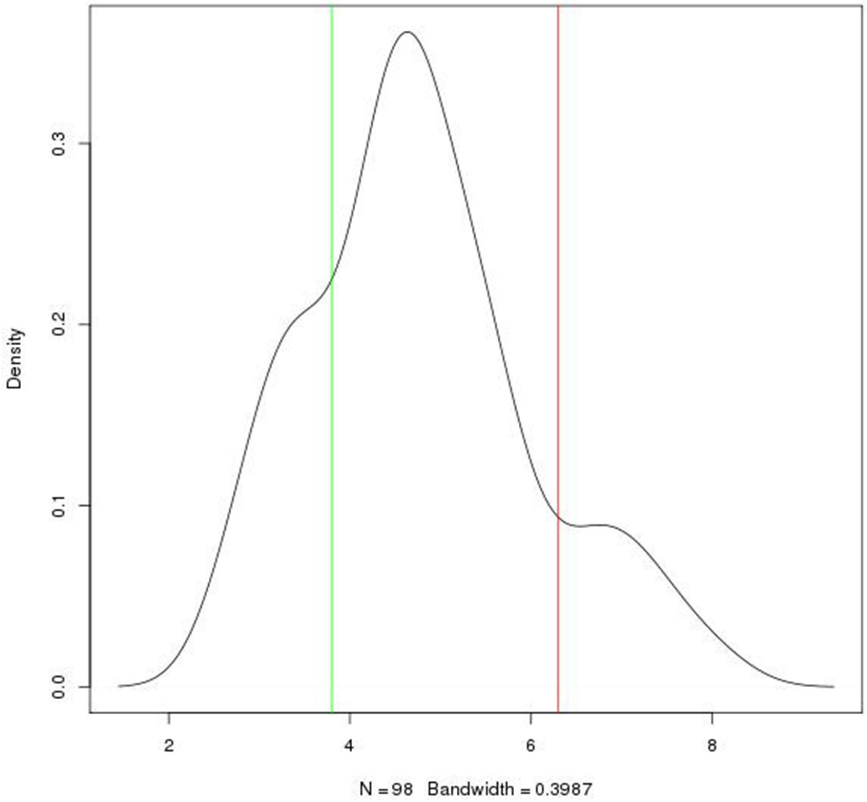

Supplementary Figure 5: Density plot showing *CD8A* level of expression.

Supplementary Table 1: Functional analysis.

See Supplementary File 1

Supplementary Table 2: Baseline characteristics of CRC patients

**CRC patients exome sequencing (n=42)**	
Gender	
Male	31 (73,8 %)
Female	11 (26,2 %)
Median age (range,years)	70 (43 - 84)
Site	
Right	12 (28,6 %)
Left	30 (71,4 %)
Stage	
II A	38 (90,5 %)
II B	4 (9,5 %)
Recurrence	
No relapse	21 (50 %)
Relapse	21 (50 %)
Recurrence-free median time (range,months)	60,9 (6,8 – 127,8)
